# Phytochemical, Antimicrobial, Insect-Repellent, and Molecular Docking Profiles of Gamma-Irradiated *Cymbopogon citratus* Essential Oil

**DOI:** 10.3390/microorganisms14071417

**Published:** 2026-06-28

**Authors:** Jaber Maataoui, Bahia Abdelfattah, Houssam Annaz, Oussama Khibech, Amr Kchikich, Amena Mrabet, Mbarek Ouabou, Abdelaaty A. Shahat, Rashed N. Herqash, Joe Miantezila Basilua, Amal El Amrani, Mohamed Khaddor

**Affiliations:** 1 Laboratory of Materials, Natural Substances and Environment (LAMSE), Chemistry Department, Faculty of Sciences and Techniques of Tangier, Abdelmalek Essaadi University, Tangier 90000, Morocco; 2 Research Laboratory Biology, Environment, and Sustainable Development, National Higher School (ENS), Abdelmalek Essaadi University, Tétouan 93000, Morocco; 3 Laboratory of Applied and Environmental Chemistry (LCAE), Department of Chemistry, Faculty of Sciences, Mohammed Premier University, Oujda 60000, Morocco; 4 Laboratory of Biotechnological Valorization of Microorganisms, Genomics, and Bioinformatics, Faculty of Sciences and Techniques, Abdelmalek Essaadi University, Tangier 90000, Morocco; 5 Department of Pharmacognosy, College of Pharmacy, King Saud University, Riyadh 11451, Saudi Arabia; 6 Center for Research on Medicinal, Aromatic, and Poisonous Plants, DSR, King Saud University, Riyadh 11451, Saudi Arabia; 7 Department of Biostatistics and Mathematics, Faculty of Pharmacy, Paris Cité University, 4 Avenue de l’Observatoire, 75006 Paris, France

**Keywords:** *Cymbopogon citratus*, gamma irradiation, essential oil, citral, antimicrobial activity, insect repellency, molecular docking

## Abstract

Gamma irradiation is one of the techniques widely authorized for the decontamination of dried herbs and spices. Its effect on the functional properties of essential oils, however, remains incompletely characterized. In this study, we examined the impact of gamma irradiation (at 5, 15, and 25 kGy) on the phytochemical composition, antimicrobial activity, antioxidant capacity, and insect-repellent activity of *Cymbopogon citratus* essential oil. The GC-MS analysis revealed that the citral-dominant chemotype remained stable across all irradiation doses, with geranial and neral constituting approximately 62–63% of the volatile profile. The antibacterial assays were done on five bacterial strains (*Staphylococcus aureus*, *Bacillus subtilis*, *Streptococcus* spp., *Pseudomonas aeruginosa*, and *Klebsiella pneumoniae*). Inhibition zones showed no statistically significant differences across irradiation doses (*p* ≥ 0.05), while MIC (75–100 µg/mL) and MBC (125–150 µg/mL) values remained constant across all doses. DPPH, ABTS, and FRAP antioxidant assays revealed no dose-dependent changes (DPPH IC_50_: 688–703 µg/mL; ABTS IC_50_: 18–22 µg/mL; FRAP: 505–517 µg/mL ascorbic-acid equivalents). The essential oil exhibited pronounced repellent activity (87–99%) against adult *Tribolium confusum* beetles at 0.125 µL/cm^2^, persisting for 24 h and unaffected by irradiation. Molecular docking of the major constituents (geranial, neral, geraniol, and β-myrcene) against key target proteins (3N7H, 3NVY, 4URM, and 8BN6) provided predictive support consistent with the observed activities, indicating plausible molecular interactions rather than confirmed target engagement. In silico ADME and toxicity profiling indicated favorable predicted pharmacokinetic properties and no major in silico toxicity alerts for the four modeled constituents. Taken together, these findings indicate that, under the conditions tested, gamma irradiation at food-decontamination doses produced no major shifts in composition and no statistically detectable changes in the measured bioactivities of *C. citratus* essential oil.

## 1. Introduction

Aromatic and medicinal plants and their essential oils (EOs) are integral to global food systems. However, they are frequently vulnerable to microbial contamination and storage pests. These problems reduce product quality, create food-safety hazards, and cause economic losses across grain and herbal value chains [1,2]. Against this background, plant-derived EOs are increasingly considered greener antimicrobial and insect-management tools. They act through multiple modes of action, degrade rapidly in the environment, and can reduce reliance on synthetic pesticides [3,4,5]. In food-preservation systems, microbial spoilage and stored-product insect infestation often occur together; an ideal botanical protectant should therefore combine antimicrobial and insect-repellent functions, which makes the simultaneous evaluation of both activities particularly relevant. Lemongrass (*Cymbopogon citratus*) EO is one of the most commonly studied botanicals: its citral-dominant chemotype (geranial + neral) is usually the major component of the volatile fraction and the basis for broad-spectrum antimicrobial, antioxidant, and insecticidal/repellent activities [6,7,8].

Ionizing irradiation is an approved post-process decontamination step for foods and ingredients such as dried herbs and spices. U.S. regulations currently permit treatment of spices, seasonings, and vegetable substances at doses up to 30 kGy, whereas the EU framework permits irradiation of dried aromatic herbs and spices at doses up to 10 kGy [9,10,11]. When used correctly, irradiation reduces microbial and pathogen loads and insect infestation without inducing radioactivity or unsafe residues [9,11]. The 5–25 kGy range examined in the present study was selected to bracket these regulatory and industrial decontamination limits: 5 kGy reflects the lower, sub-regulatory doses commonly applied to herbs, the EU ceiling of 10 kGy falls within this bracketed range, and 15 and 25 kGy probe the upper decontamination range tolerated under the U.S. framework and applied industrially to heavily contaminated lots. However, because ionizing radiation can generate reactive radical species—chiefly through the radiolysis of residual water and the direct ionization of organic molecules—it may, in principle, promote cis–trans isomerization between the citral isomers (neral and geranial), oxidation of the reactive aldehyde function toward geranic acid, and the formation of secondary oxygenated derivatives. It is therefore important to verify that citral-rich EOs retain their composition and bioactivity after decontamination-level treatment [12,13].

Recent data show that moderate gamma irradiation doses (≤25 kGy) generally leave EO chemotypes and functional activities largely unchanged, with only slight and matrix-specific changes in a few monoterpenes or oxygenated sesquiterpenes [12,13]. Low doses have also been reported to enhance the phenolic content or antioxidant readout in other aromatic plants, although species- and assay-specific effects have been observed [14]. In the case of *C. citratus* in particular, chemical profiling today confirms citral as the main contributor to antibacterial and antioxidant activity, providing a mechanistic rationale to anticipate functional stability post-decontamination-level irradiation [6,8].

In addition to microbiological safety, stored-product insects (e.g., *Tribolium* spp., *Sitophilus* spp.) cause chronic postharvest losses and degrade the sensory and nutritional quality of stored commodities. Lemongrass EO and its main constituents (citral isomers, geraniol) show pronounced repellency and contact/fumigant toxicity against these pests [15,16]. More broadly, their monoterpene-rich volatile fractions act as eco-friendly insecticides through multiple complementary modes of action against both stored-product and field pests [17,18]. Whether decontamination-level gamma irradiation alters this repellent performance in *C. citratus* EO remains insufficiently established [19], even though individual monoterpenes characteristic of lemongrass oil are increasingly recognized as effective green insecticides against stored-product beetles [20,21].

Even though irradiation is widely authorized for dried spices, there is still a lack of systematic analyses that combine (i) detailed GC-MS chemotyping of *C. citratus* EO with (ii) antimicrobial activity (zones, MIC/MBC), (iii) antioxidant activity (DPPH, ABTS, FRAP) and (iv) insect-repellent activity in stored-product beetles across gamma irradiation dose gradients. Available data on other EOs indicate compositional stability and retained antimicrobial action at decontamination doses [12,13], but comprehensive datasets remain limited, particularly those that extend to the endpoint of repellency in *Tribolium* spp. (especially during extended exposure periods) [17,19].

This research paper explores the hypothesis that gamma irradiation within food-processing-relevant ranges may modulate the phytochemical, antimicrobial, antioxidant, and insect-repellent properties of *Cymbopogon citratus* EO. In particular, we (1) profiled EO composition by GC-MS; (2) measured antibacterial activity against Gram-positive and Gram-negative strains (zones of inhibition, MIC, MBC); (3) measured antioxidant capacity by DPPH, ABTS, and FRAP; and (4) measured repellency of adult *Tribolium confusum* at effective surface doses at different exposure times, comparing non-irradiated with gamma-irradiated samples (5–25 kGy). The integrated design directly addresses whether the functional hallmarks of lemongrass EO that are important in food protection applications are retained under decontamination-level irradiation.

## 2. Materials and Methods

### 2.1. Plant Material

Harvesting of fresh aerial portions of *Cymbopogon citratus* (lemongrass) took place in the Tangier region (northern Morocco) in the early vegetative phase (April–May). Post-harvest, the material was checked to eliminate debris and epiphytes and then dried in a single layer at 22 ± 2 °C and 45–55% relative humidity in darkness to minimize enzymatic degradation and to preserve the volatiles [22,23]. Light-proof, food-grade bags containing the dried biomass were stored at 4 °C until irradiation.

### 2.2. Gamma Irradiation

The dried plant material was irradiated using a ^60^Co irradiator (industrial panoramic source) in Aragogamma, a licensed facility (Barcelona, Spain). Sample sizes of approximately 300 g were put in perforated polyethylene containers to allow uniform penetration of doses. Three nominal doses were used (5, 15 and 25 kGy) and also a non-irradiated control (0 kGy), with three independent replicates per dose (*n* = 3). Dose mapping with alanine dosimeters confirmed ±10% dose uniformity across containers. All the runs were performed at ambient temperature (~25 °C) in the dark to reduce thermo-oxidative effects [11,24,25]. After the treatment, the samples were returned to cold, dark storage (4 °C) until extraction.

### 2.3. Essential Oil Extraction

The hydrodistillation was conducted after irradiation to determine the changes in the volatile profile due to the treatment. To each lot, 250–300 g of cut and dried aerial parts were distilled for 3 h with distilled water (1:4–1:5 *w*/*v*) using a Clevenger-type apparatus (pharmacopeial method) [23,26]. The essential oil (EO) layer was transferred to amber, crimp-sealed glass vials (1.5 mL), minimizing headspace. Vials were kept at 4 °C in the dark to reduce the effect of photo- and auto-oxidation before analysis [27,28]. For each lot, the EO yield was determined gravimetrically in duplicate (*n* = 2) and expressed as a percentage (*w*/*w*) relative to the dry weight of the distilled biomass. To minimize post-irradiation aging, hydrodistillation was carried out within 7 days of irradiation, and all biological assays were completed within 4 weeks of extraction; between steps, both the dried biomass and the extracted EO were stored at 4 °C in the dark.

### 2.4. Essential Oil Analysis

#### Gas Chromatography/Mass Spectrometry (GC-MS) Analysis

Volatile compounds were analyzed by GC-MS on a Thermo Fisher Scientific TRACE 1300 GC coupled with a TSQ 8000 Evo mass spectrometer (Thermo Fisher Scientific, Waltham, MA, USA) (electron impact, 70 eV). Separation was performed using a DB-5MS (5% phenyl-95% dimethylpolysiloxane) capillary column (30 m × 0.25 mm i.d., 0.25 µm film) (Agilent J&W, Santa Clara, CA, USA). The oven program was 60 °C (2 min), followed by a ramp of 3 °C/min to 180 °C and 5 °C/min to 280 °C (10 min hold). Helium was used as the carrier gas at 1.2 mL/min. The injection conditions were split 1:20, 0.8 µL injection volume, EO in hexane (n-hexane, ≥95%, Sigma-Aldrich, St. Louis, MO, USA) (~1% *v*/*v*), and injector temperature 250 °C. The MS source and transfer-line temperatures were 250 °C and 280 °C, respectively, with scanning from m/z 40 to 700 at ~5 scans/s. Compound identification was based on comparison of EI spectra and linear retention indices (LRIs) with the NIST/EPA/NIH libraries and authenticated references; LRIs were calculated relative to a C8–C25 n-alkane series analyzed under the same chromatographic conditions [29,30]. Assignments were retained only when the mass spectrum matched reference/library spectra and the experimental LRI differed from the selected literature LRI by not more than 10 index units for the DB-5-type non-polar column. Semi-quantification was performed by normalized area percentage without response-factor correction. The experimentally calculated LRI values, the corresponding literature LRIs, and ΔLRI values (experimental minus literature) are reported in Table 1 as rounded integer indices; the underlying n-alkane calibration worksheet was retained with the source data. GC-MS analysis was performed as a single injection per irradiation dose; only peaks with a signal-to-noise ratio ≥ 3 were retained. Very low-abundance constituents (<0.2%) are reported as trace detections only; because replicate GC-MS injections were not available, their cross-replicate consistency could not be assessed, and they were not used to infer irradiation-dependent trends.

### 2.5. Antibacterial Testing

#### 2.5.1. Preparation of the Bacterial Suspension

The bacteria tested comprised five strains (four ATCC reference strains and one clinical isolate): *Staphylococcus aureus* (ATCC 25923), *Bacillus subtilis* (ATCC 6633), *Pseudomonas aeruginosa* (ATCC 27853), *Klebsiella pneumoniae* (ATCC 700603), and *Streptococcus* spp. clinical isolate. According to the current guidelines, stock cultures were sub-cultured on Mueller–Hinton agar (MHA) and cultivated in cation-adjusted Mueller–Hinton broth (CAMHB) to the 0.5 McFarland standard (~1 × 10^8^ CFU/mL) [31,32].

#### 2.5.2. Agar Disk Diffusion

The standardized suspensions were inoculated (100 µL, evenly spread) on pre-poured MHA plates (approximately 20 mL/plate). EO solutions (control 0 kGy, and 5, 15, 25 kGy lot; fixed loading per disk) were impregnated on sterile 6 mm paper disks (Whatman No. 1, Cytiva, Marlborough, MA, USA) and placed on the agar (≤6 disks/plate) and pre-diffused for 15 min at room temperature and then incubated (37 °C, 18–24 h). Calibrated digital calipers/automated reader were used to measure inhibition-zone diameters (mm). The conditions were run in quadruplicate (*n* = 4) [31,33]. Gentamicin (10 µg) disks (Bio-Rad, Marnes-la-Coquette, France) served as positive controls for the Gram-negative strains (*P. aeruginosa*, *K. pneumoniae*), and vancomycin (30 µg) disks (Bio-Rad, Marnes-la-Coquette, France) for the Gram-positive strains (*S. aureus*, *B. subtilis*, *Streptococcus* spp.); disks loaded with solvent alone served as negative controls. The ATCC reference strains produced inhibition zones consistent with their expected susceptibility profiles; for the *Streptococcus* spp. clinical isolate, the vancomycin control confirmed inhibition under the assay conditions but was not assigned a categorical breakpoint interpretation. The antibiotic disks were included only as reference growth-inhibition (assay validity) controls confirming strain viability and inhibition by a known reference agent under the test conditions; they were not used for CLSI/EUCAST breakpoint categorization, and vancomycin in particular is reported here solely as a growth-inhibition control and not as a disk-diffusion susceptibility test.

#### 2.5.3. Determination of the Minimum Inhibitory and Minimum Bactericidal Concentrations

The 96-well broth microdilution in CAMHB was used to determine MICs according to CLSI M07-A11 principles with modifications to accommodate hydrophobic EOs (≤1% final solvent/emulsifier; 0.5% Tween-80 (Sigma-Aldrich, St. Louis, MO, USA) or 0.5% DMSO (dimethyl sulfoxide, ≥99.5%, Sigma-Aldrich, St. Louis, MO, USA) as needed). Test concentrations spanned the 25–200 µg/mL range. Wells were inoculated at a concentration of approximately 5 × 10^5^ CFU/mL and incubated at 37 °C for 18–24 h. The growth was measured by colorimetry using resazurin (0.015%; Sigma-Aldrich, St. Louis, MO, USA); MIC was the lowest concentration that avoided the color change/visual growth. For MBC, 10–20 µL from non-turbid wells were plated onto MHA and incubated; the MBC was the lowest concentration yielding no colonies [31,34,35].

### 2.6. Antioxidant Assays

Complementary radical-scavenging (DPPH, ABTS•+) and electron-transfer (FRAP) assays were used to measure antioxidant capacity of irradiated and control EOs (0, 5, 15, 25 kGy) to identify various mechanisms of action [36,37]. Measurements were done in quadruplicate (*n* = 4) and the results were reported as IC_50_ (DPPH/ABTS•+) or equivalent antioxidant capacity (FRAP).

#### 2.6.1. DPPH Radical-Scavenging Assay

DPPH (2,2-diphenyl-1-picrylhydrazyl, Sigma-Aldrich, St. Louis, MO, USA) (200 µM) was incubated with EO dilutions (methanol, ≥99.9%, Sigma-Aldrich, St. Louis, MO, USA; 0.25–10 µL/mL) in the dark at room temperature (1:1). The absorbance was measured at 517 nm in comparison to reagent blanks; dose–response curves were used to determine percent inhibition and IC_50_ [36,38].

#### 2.6.2. ABTS•+ Decolorization Assay

The ABTS•+ radical cation was prepared by reacting 7 mM ABTS (2,2′-azino-bis(3-ethylbenzothiazoline-6-sulfonic acid), Sigma-Aldrich, St. Louis, MO, USA) with 2.45 mM K_2_S_2_O_8_ (potassium persulfate, Sigma-Aldrich, St. Louis, MO, USA) in the dark during a period of 12–16 h and then diluted with ethanol (≥99.8%, Sigma-Aldrich, St. Louis, MO, USA) to A_734_ = 0.70 ± 0.02. Two milliliters of ABTS•+ solution were mixed with 100 µL of EO, and after 6 min, A_734_ was measured and IC_50_ calculated [37,39].

#### 2.6.3. Ferric Reducing Antioxidant Power (FRAP) Assay

FRAP reagent (acetate buffer 300 mM, TPTZ (2,4,6-tris(2-pyridyl)-s-triazine, Sigma-Aldrich, St. Louis, MO, USA) 10 mM in HCl 40 mM, FeCl_3_ (iron(III) chloride, Sigma-Aldrich, St. Louis, MO, USA) 20 mM; 10:1:1) was prepared fresh and equilibrated at 37 °C. EO aliquots (100–300 µL) were mixed with 3.0 mL FRAP reagent; after 4–6 min, absorbance was read at 593 nm. Results were expressed as ascorbic-acid (L-ascorbic acid, ≥99%, Sigma-Aldrich, St. Louis, MO, USA) equivalents from calibration curves [36,38,40].

### 2.7. Insecticidal and Repellent Assays

#### 2.7.1. Insect Colony

Adults (7 to 21 days old) of *Tribolium confusum* J. du Val 1863 were obtained from infested flour. The identity was confirmed using a binocular microscope. Insects were reared under laboratory conditions in a mixture of flour and yeast (19:1) *w*/*w*. Insects were maintained in an incubator at a temperature of 30 °C and a humidity of 65 ± 5% in the dark.

#### 2.7.2. Repellent Bioassay

The repellent effect of essential oils was assessed according to the method of McDonald et al. [41]. The bioassay was conducted in two stages. In the first stage, the non-irradiated EO was screened at three concentrations (0.2, 0.4, and 0.8% *v*/*v* in acetone, corresponding to surface doses of 0.031, 0.062, and 0.125 µL/cm^2^ on the treated half-disk) to establish the concentration–response relationship and to identify the most effective operative concentration. In the second stage, the irradiated EOs were tested at this single most effective concentration. This sequential design was adopted deliberately: by fixing the operative concentration at the level relevant for practical protection, the comparison isolates the effect of irradiation itself—rather than of concentration—on repellency, while limiting the number of insect-consuming bioassays. We acknowledge that testing every irradiation group across the full concentration range would provide a more exhaustive description of dose×concentration interactions, and this is noted as a direction for future work (Section 4.4). Practically, filter paper (Whatman No. 1, Cytiva, Marlborough, MA, USA) (7 cm in diameter) was divided into two halves, where one received 300 µL of a given concentration while the other received the same volume of pure acetone (≥99.5%, Sigma-Aldrich, St. Louis, MO, USA).

Treated halves were left at room temperature for 3 min to allow the acetone to evaporate. The two halves were then joined with paper tape and placed in a Petri dish (7 cm diameter). Thirty unsexed adults were introduced in the middle, and the number of adults present in each half was recorded after 0.5, 1, 2, 4, and 24 h. Five replicates were conducted for each treatment. The counts were used to calculate the percentage of repellency as described by McDonald et al. [41] using the following equation:

PR = [(N_C_ − N_t_)/(N_C_ + N_t_)] × 100 (1)

where N_C_ and N_t_ are the number of adults present in the half treated with acetone and EO concentration, respectively.

### 2.8. In Silico ADME and Toxicity Prediction

The selected major constituents of *Cymbopogon citratus* essential oil (geranial, neral, β-myrcene and geraniol) were subjected to an integrated in silico ADMET evaluation. For each molecule, the canonical SMILES string was obtained from PubChem and used without modification as the input format for all predictive platforms. SwissADME (www.swissadme.ch, accessed on June 2026) was used to estimate physicochemical descriptors, lipophilicity, aqueous solubility, drug-likeness filters, oral bioavailability radar parameters and BOILED-Egg permeability behavior. Complementary pharmacokinetic endpoints, including human intestinal absorption, blood–brain barrier permeability, central nervous system permeability, cytochrome P450-related liabilities, renal OCT2 transport and total clearance, were predicted using pkCSM (https://biosig.lab.uq.edu.au/pkcsm, accessed on June 2026). Toxicological endpoints were evaluated using ADMET-AI (https://admet.ai.greenstonebio.com, accessed on June 2026), with particular attention to acute toxicity LD_50_ and clinical toxicity probability in relation to the DrugBank reference chemical space [42,43,44,45]. The combined workflow provided a rapid comparative assessment of absorption, distribution, metabolism, excretion and preliminary safety liabilities for the principal bioactive volatiles before docking-based mechanistic analysis.

### 2.9. Molecular Docking Protocol

**Figure 1:**
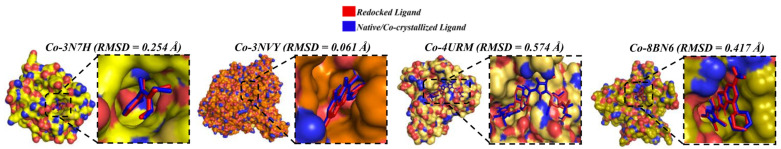
Validation of the molecular docking protocol by redocking the native/co-crystallized ligands into the binding sites of 3N7H, 3NVY, 4URM, and 8BN6. Redocked ligands are shown in red and native/co-crystallized ligands in blue; the low RMSD values (Co-3N7H, RMSD = 0.254 Å; Co-3NVY, RMSD = 0.061 Å; Co-4URM, RMSD = 0.574 Å; Co-8BN6, RMSD = 0.417 Å) confirm reliable reproduction of the crystallographic binding modes.

Molecular docking was performed using AutoDock Vina v1.2.0 implemented in PyRx v0.9 [44,46,47,48]. The crystal structures of the selected targets were retrieved from the Protein Data Bank and comprised 3N7H, an insect odorant-binding protein model co-crystallized with a repellent ligand; 3NVY, bovine xanthine oxidase in complex with quercetin; 4URM, *Staphylococcus aureus* DNA gyrase B ATPase domain in complex with kibdelomycin; and 8BN6, *Pseudomonas aeruginosa* DNA gyrase B ATPase domain in complex with EBL3021. Receptor preparation was performed in AutoDockTools v1.5.7 by removing crystallographic water molecules and non-essential heteroatoms, adding polar hydrogens, assigning receptor charges and converting the prepared structures into PDBQT format. Co-crystallized ligands were extracted from their binding sites and used for redocking validation, while structurally relevant cofactors or ions were retained when required to preserve the local binding-site architecture. Ligand structures were energy-minimized, protonated in their neutral forms when appropriate and converted to PDBQT with rotatable bonds defined before docking. Docking reliability was evaluated by re-docking the native/co-crystallized ligand into each crystallographic site using an exhaustiveness value of 20 and grid boxes centered on the corresponding experimental binding pockets as follows: 3N7H, center (x = 13.91, y = 4.40, z = 14.00) and size (x = 8.62, y = 12.35, z = 7.10 Å); 3NVY, center (x = 39.60, y = 21.95, z = 20.19) and size (x = 10.81, y = 10.86, z = 12.56 Å); 4URM, center (x = 40.28, y = −1.07, z = 28.13) and size (x = 14.93, y = 13.64, z = 25.28 Å); and 8BN6, center (x = −2.43, y = 22.94, z = 0.60) and size (x = 13.80, y = 12.10, z = 12.41 Å). The redocked poses reproduced the experimental orientations with RMSD values of 0.254 Å for Co-3N7H, 0.061 Å for Co-3NVY, 0.574 Å for Co-4URM and 0.417 Å for Co-8BN6 (Figure 1), demonstrating that the protocol was sufficiently accurate for subsequent pose prediction and interaction analysis. Molecular visualization and figure preparation were carried out using PyMOL v2.5 (Schrödinger, LLC, New York, NY, USA) and BIOVIA Discovery Studio 2024 (Dassault Systèmes, Vélizy-Villacoublay, France).

### 2.10. Statistical Analysis

Antibacterial (disk-diffusion) and antioxidant assays were performed in quadruplicate (*n* = 4), repellency assays in five replicates (*n* = 5), and EO yield in duplicate technical determinations (*n* = 2). Continuous data (inhibition-zone diameters and antioxidant IC_50_/FRAP values) were analyzed by one-way ANOVA followed by Tukey’s multiple range test (SAS, version 9.4; SAS Institute Inc., Cary, NC, USA) and are expressed as mean ± standard deviation, except for repellency data, which are expressed as mean ± standard error. MIC and MBC endpoints were summarized as modal values across replicates and were not subjected to parametric testing. Because the EO yield was measured only as duplicate technical determinations, it was compared descriptively only. GC-MS analysis was performed as a single injection per dose. Differences were considered significant at *p* < 0.05. To examine overall profile similarity, the GC-MS area-% data were subjected to exploratory hierarchical cluster analysis (HCA, average linkage, Euclidean distance) after row-standardization of each constituent, and pairwise Pearson correlations between dose profiles were computed; the heatmap and dendrogram are presented in the Supplementary Materials (Figure S1). Because GC-MS replicate injections were unavailable, replicate-based multivariate models such as PCA or PLS-DA were not statistically appropriate. Accordingly, the HCA is used only as an illustrative visualization of single-injection dose profiles, not as inferential evidence for treatment separation.

## 3. Results

### 3.1. Chemical Composition

The EO yield was stable across the irradiation range. Duplicate technical determinations gave 0.92 ± 0.02% for the non-irradiated control (individual values: 0.93 and 0.90%), 0.90 ± 0.01% at 5 kGy (0.91 and 0.89%), 0.93 ± 0.01% at 15 kGy (0.92 and 0.94%), and 0.89 ± 0.01% at 25 kGy (0.89 and 0.88%) (*w*/*w* relative to dry biomass). Because these were technical duplicate measurements from each dose lot, the yield data were compared descriptively only and were not used for inferential statistics. The GC-MS profile of Cymbopogon citratus essential oil (Table 1) remained citral-dominant across all treatments. Geranial and neral were the main constituents and together accounted for about 63% in the control and about 62% at the highest dose, indicating preservation of the chemotype. β-myrcene was the leading hydrocarbon and showed a small, gradual decline, while geraniol increased from roughly 5% to slightly above 6%. Minor oxygenated compounds rose slightly with dose, including caryophyllene oxide, acetophenone, eucalyptol, 2-undecanone, 2-tridecanone, and carveol, whereas limonene, citronellal, and the formate esters remained low-abundance constituents. These changes were small relative to the dominance of the citral isomers and should be interpreted cautiously because GC-MS replicate injections were not available.

### 3.2. Antimicrobial Activity

#### 3.2.1. Effect of Gamma Irradiation on Antibacterial Activity (Inhibition Zones)

*Cymbopogon citratus* essential oil was not significantly affected by gamma irradiation at doses ranging from 5 to 25 kGy, as this treatment did not affect its antimicrobial activity against Gram-positive and Gram-negative bacteria (*p* ≥ 0.05). The inhibition zones were similar to those of the non-irradiated control, indicating that the structural integrity and biological activity of the oil’s antibacterial constituents remained largely unaffected by ionizing radiation. As commonly reported, Gram-positive strains were more susceptible than Gram-negative ones; however, no irradiation-dependent variation was observed either within or among the tested microorganisms, as presented in Table 2. The reference antibiotics (gentamicin and vancomycin) produced the expected inhibition zones, confirming assay validity. A direct potency comparison between the essential oil and the reference antibiotics was not made because of differences in loading, composition, and diffusion behavior on agar.

#### 3.2.2. Minimum Inhibitory Concentration (MIC)

There was no noticeable difference in the minimum inhibitory concentration (MIC) of *Cymbopogon citratus* essential oil following gamma irradiation at doses between 5 and 25 kGy. The MIC values remained almost identical across the irradiation treatments, showing that the antimicrobial activity of the oil was not influenced by exposure to ionizing radiation. As expected, Gram-positive bacteria (*Staphylococcus aureus*, *Bacillus subtilis*, and *Streptococcus* spp.) required lower concentrations to inhibit growth (75 µg/mL) than Gram-negative species (*Pseudomonas aeruginosa* and *Klebsiella pneumoniae*, 100 µg/mL). These results confirm that the antibacterial effect of *C. citratus* oil stayed unchanged after irradiation, as shown in Table 3.

#### 3.2.3. Minimum Bactericidal Concentration (MBC)

*Cymbopogon citratus* essential oil exposed to gamma irradiation doses of 5 to 25 kGy did not exhibit any apparent difference in the minimum bactericidal concentration (MBC). The MBC values were not affected by the irradiation treatment in any of the bacterial strains tested, suggesting that ionizing radiation did not affect the bactericidal effect of the oil. The Gram-positive bacteria (*Staphylococcus aureus*, *Bacillus subtilis*, and *Streptococcus* spp.) had lower MBC values (125 µg/mL) than Gram-negative species (*Pseudomonas aeruginosa* and *Klebsiella pneumoniae*, 150 µg/mL). These results confirm that the bactericidal effect of *C. citratus* oil did not change after being subjected to irradiation, as indicated in Table 4.

### 3.3. Antioxidant Activity

The antioxidant activity (IC_50_, µg/mL) of *Cymbopogon citratus* essential oil subjected to different doses of gamma irradiation (5–25 kGy) did not differ significantly (*p* ≥ 0.05). The DPPH, ABTS, and FRAP assays revealed similar values across the various irradiation levels, showing that exposure to ionizing radiation did not affect the radical-scavenging capacity or the reducing capacity of the oil. In general, the antioxidant activity of *C. citratus* was not dependent on the dose applied, as indicated by the results in Table 5.

### 3.4. Repellent Activity

#### 3.4.1. Effect of Concentration and Exposure Time

*Cymbopogon citratus* EO displayed strong repellency against adults of *T. confusum*, depending on the concentration (*p* < 0.05) but not the exposure time (*p* > 0.05) as indicated in Table 6. The highest repellency was observed at the highest concentration (0.125 µL/cm^2^), where the percentage of repellency ranged between 89 and 95% over 24 h of experiment. The highest PR observed was 95% at this concentration after 2 h.

#### 3.4.2. Effect of Gamma Irradiation on Repellent Activity

**Figure 2:**
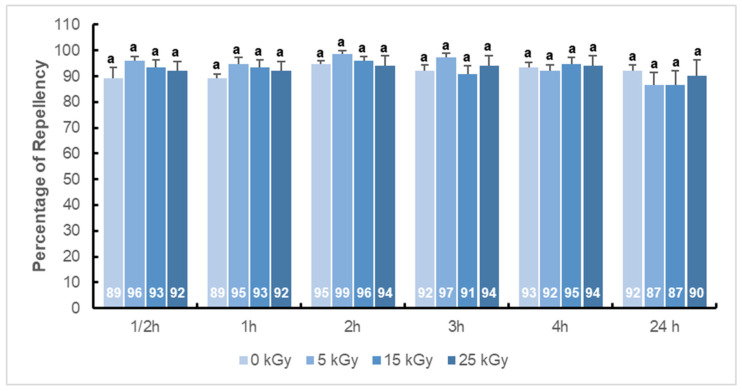
Repellent effect of *Cymbopogon citratus* essential oil irradiated at different doses, tested at the most efficient concentration (0.125 µL/cm^2^) against adults of *Tribolium confusum*. Values are expressed as the mean of the percentage of repellency (PR) ± standard error for five replicates using 30 adults each. Results are considered significantly different when the lowercase and uppercase letters differ at *p* < 0.05 across irradiation doses and exposure durations, respectively. Error bars represent the standard error (SE) of the mean. Bars sharing the same letter are not significantly different, because gamma irradiation did not significantly affect repellency; all doses share the letter “a” at each exposure time, which is the intended outcome of the analysis.

Regarding the effect of irradiation on the repellent activity of *C. citratus* EO, no significant difference was observed between the different doses (F = 0.3, *p* = 0.794) at various exposure durations (F = 2.3, *p* = 0.066). In terms of effectiveness, EO irradiated with 5 kGy exhibited the highest level of repellency during the early exposure period (0.5–2 h), with the highest PR (99%) recorded after 2 h, as shown in Figure 2. After 24 h, the PR levels slightly decreased but remained within the range of 87–92%.

## 4. Discussion

### 4.1. Chemical Composition

The citral-dominant chemotype of *Cymbopogon citratus* essential oil was retained after gamma irradiation (5–25 kGy), and geranial and neral were the major constituents, with only slight changes in minors (e.g., a slight decrease in β-myrcene and a slight increase in geraniol and oxygenated sesquiterpenes). This trend is consistent with recent reports stating that lemongrass oil is inherently high in citral isomers and shows little compositional change with processing [49,50]. Furthermore, studies monitoring irradiation of essential oils have shown that moderate gamma irradiation doses rarely cause major compositional shifts, most often yielding minor oxidation or isomerization without reducing the abundance of key oxygenated monoterpenes, attributable to the stability of major volatiles at ≤25 kGy [12,13]. The compositional stability observed here helps explain the unchanged antimicrobial, antioxidant, and repellent activities, because citral and other oxygenated monoterpenes are the major drivers of these bioactivities in lemongrass oils [51,52].

### 4.2. Antimicrobial Activity

Gamma irradiation of *Cymbopogon citratus* essential oil (EO) at 5–25 kGy did not measurably affect antibacterial action across the zones of inhibition, MIC, and MBC. This stability is in line with recent studies indicating that when gamma irradiation is used for decontamination, EO antibacterial activity largely persists, with only slight chemical profile changes depending on matrix and dose [12,53]. The Gram-positive versus Gram-negative susceptibility pattern also matches the citral-dominated chemotype of lemongrass EO, which is rich in geranial and neral, shows intrinsic antimicrobial activity, and remains active under moderate processing conditions [6]. Other EOs at similar dose ranges (e.g., ylang-ylang) showed no meaningful pre-/post-irradiation differences in activity, supporting the conclusion that the key antibacterial constituents are stable at these doses [54].

Mechanistically, low irradiation doses can promote limited isomerization or oxidation of monoterpenes without loss of overall activity, effects that can be chemotype-specific rather than uniformly detrimental [12,53]. The lack of an irradiation-dependent trend in our MIC and MBC data is consistent with citral isomers and related oxygenated monoterpenes remaining within an active range despite exposure.

### 4.3. Antioxidant Activity

DPPH, ABTS, and FRAP showed no significant change in antioxidant capacity at 5–25 kGy, indicating an overall neutral effect of irradiation. In other botanicals, low-to-moderate gamma irradiation doses can either increase antioxidant capacity or leave it unchanged, depending on phenolic release versus degradation and assay chemistry [14,55,56]. Our neutral result fits within those reports, especially since some improvements elsewhere occur below 5–7.5 kGy and might be missed when 5 kGy is the starting dose [14,56].

Regardless of irradiation, the antioxidant activity of lemongrass EO is generally moderate compared with phenolic-rich EOs, which aligns with our IC_50_ ranges and modern descriptions of citral-rich chemotypes [6,8]. Differences between studies likely reflect cultivar, extraction method, and assay conditions (radical type, solvent system).

### 4.4. Insecticidal and Repellent Activity

Our results highlighted a strong repellent potential of *C. citratus* EOs against adults of *T. confusum* lasting for over 24 h. This persistent repellency over 24 h has been reported less frequently, as most previous studies examined only short-term exposure (1–5 h). This strong repellency can be attributed to the richness in oxygenated monoterpenoids (neral and geranial) and monoterpene hydrocarbons (β-myrcene). When tested individually, geranial strongly repelled two stored-product insects, *Callosobruchus maculatus* and *Sitophilus zeamais* [57]. Other EOs with similar or close chemical profiles have been documented for their strong repellency against stored-product insects. For instance, geraniol- and nerol-rich EOs obtained from *C. citratus* and *Melissa officinalis* were documented for their strong repellent activity against *Tribolium castaneum* [58,59]. Moreover, *Lippia alba* EOs with almost similar chemical composition (24.5 and 11.9% of geranial and neral, respectively) exhibited strong repellency against adults of *Ulomoides dermestoides* [60]. Lemongrass EO rich in geraniol (20.86%) and limonene (10.5%) exhibited a strong repellent effect against *Sitophilus granarius* with PR of 91.49 and 97.30% at 40 and 50 µL/mL, respectively, after 2 h of exposure [61].

Regarding the impact of irradiation on the repellent effectiveness of *C. citratus*, no significant difference was observed. This suggests that, despite possible modifications caused by the treatment, the efficacy remained unchanged. This finding contrasts with studies on *Trogoderma granarium* larvae using irradiated *Thuja orientalis* at low doses (1, 3, and 5 kGy) over a 2-hour exposure. The difference likely stems from the significant chemical composition alterations reported in that study for *T. orientalis* EO due to irradiation. Such qualitative and quantitative changes can affect the activity of essential oils, unlike our observations, where the chemical profile remained stable. Additionally, no significant difference was found in repellency percentages between irradiated (5 kGy) and non-irradiated EOs at high concentrations (0.252 µL/cm^2^), supporting our results. These findings highlight the key role of concentration in the repellent effectiveness of both irradiated and non-irradiated EOs. A limitation of the present design is that the irradiated oils were compared only at the most effective operative concentration; testing each irradiation dose across the full concentration range would allow a more complete description of any dose×concentration interactions, and this is recommended for future studies.

### 4.5. ADMET Results

ADMET prediction is an essential step in the interpretation of the bioactivity of essential oil constituents because biological potency alone does not indicate whether a compound can reach a relevant biological interface, remain sufficiently bioavailable and avoid major pharmacokinetic or toxicological liabilities. For small volatile monoterpenes such as citral isomers, β-myrcene and geraniol, ADMET descriptors are particularly informative because the same hydrophobicity that favors membrane penetration, olfactory recognition and antimicrobial interactions may also influence solubility, blood–brain barrier permeation and toxicity. Therefore, the ADMET analysis complements the experimental antimicrobial, antioxidant and insect-repellent assays by defining the pharmacokinetic plausibility and preliminary safety profile of the major bioactive constituents [62,63,64].

**Figure 3:**
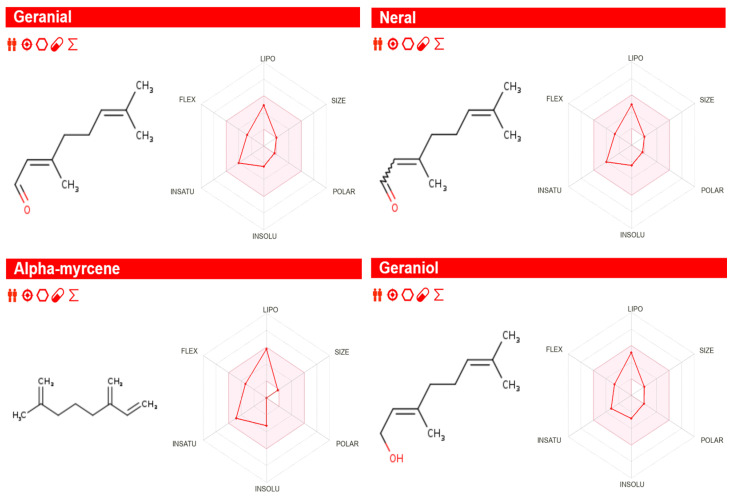
SwissADME bioavailability radar profiles and 2D chemical structures of the selected major constituents: geranial, neral, β-myrcene, and geraniol.

The SwissADME radar profiles (Figure 3) indicate that the four molecules occupy a compact monoterpenoid chemical space characterized by small molecular size, limited polarity, moderate lipophilicity and acceptable conformational flexibility. Geranial and neral show nearly superimposable radar behavior, as expected for geometric citral isomers with identical molecular weight, TPSA, hydrogen-bond acceptor count and consensus Log P [65]. Geraniol preserves the same terpenoid scaffold but introduces one hydrogen-bond donor through its hydroxyl group, resulting in the highest TPSA among the series and the most balanced polarity-solubility profile. In contrast, β-myrcene lacks heteroatoms, has TPSA = 0 Å^2^ and displays the highest lipophilicity, which explains its more hydrophobic radar signature and its less favorable SwissADME gastrointestinal absorption classification despite the high passive intestinal absorption predicted by pkCSM. Overall, Figure 3 supports the view that oxygenated monoterpenes, particularly geraniol and the citral isomers, combine membrane permeability with a minimal polar anchor, whereas β-myrcene behaves as a highly apolar terpene whose bioactivity is likely dominated by hydrophobic interactions.

Table 7 confirms that all four constituents satisfy classical oral drug-likeness filters, with no Lipinski or Veber violations and a uniform bioavailability score of 0.55. Their very low molecular weights, limited rotatable-bond counts and low TPSA values are consistent with rapid passive diffusion and explain the high pkCSM-predicted human intestinal absorption values, which remain above 92% for all molecules. The positive BBB permeability values and CNS permeability values close to the −2 threshold suggest that these terpenoids may cross biological membranes efficiently, although the more polar hydroxylated geraniol shows the lowest CNS penetration tendency. Importantly, none of the compounds is predicted to inhibit or serve as a substrate for CYP2D6 or CYP3A4, and none is predicted to be a renal OCT2 substrate, indicating a low probability of major transporter- or CYP-mediated liabilities in this preliminary model. The moderate predicted clearance values further suggest that these small volatile constituents are unlikely to accumulate extensively. Thus, the oxygenated monoterpenes display the most favorable compromise between permeability, polarity and metabolic simplicity, whereas β-myrcene is pharmacokinetically more hydrophobic and should be interpreted as a membrane-active terpene rather than a balanced oral-like molecule.

**Figure 4:**
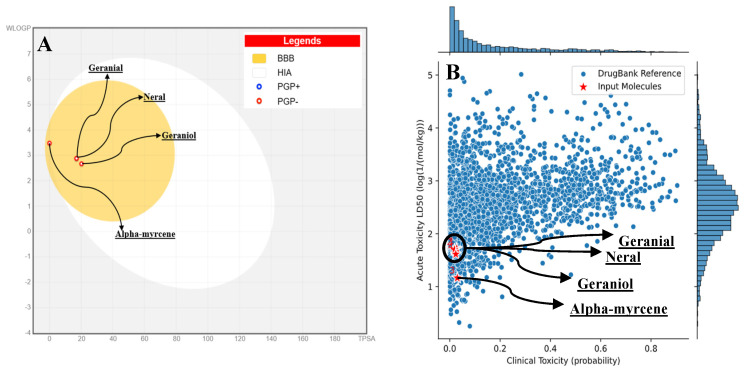
Integrated permeability and toxicity assessment. (**A**) SwissADME BOILED-Egg model showing predicted gastrointestinal absorption, blood–brain barrier permeation, and P-glycoprotein status. (**B**) ADMET-AI acute toxicity and clinical toxicity projection of the input molecules relative to DrugBank reference compounds.

The BOILED-Egg representation in Figure 4A places geranial, neral and geraniol within the high-absorption/high-permeability region, in agreement with their low TPSA and moderate lipophilicity. The red P-gp markers indicate that the studied molecules are not predicted to be P-glycoprotein substrates, suggesting that passive permeability is unlikely to be strongly counteracted by efflux [66,67]. β-myrcene occupies a more hydrophobic and minimally polar region, consistent with its high BBB permeability score and absence of hydrogen-bonding capacity. In Figure 4B, the ADMET-AI projection positions the input compounds away from the high clinical-toxicity/high acute-toxicity region of the DrugBank reference space. Geranial, geraniol and neral show a slightly higher toxicity tendency than β-myrcene, which may be related to the electrophilic aldehyde function of citral isomers, whereas geraniol benefits from a less reactive alcohol group while retaining favorable permeability. Taken together, the combined BOILED-Egg and ADMET-AI visualization indicates that the major constituents have a plausible permeability profile and no dominant in silico toxicity alarm, although experimental toxicological confirmation remains necessary before any translational claim.

### 4.6. Molecular Docking

Because gamma irradiation did not appreciably alter the chemical composition of the oil, the aim of the docking analysis was not to compare irradiated and non-irradiated samples but to provide a molecular-level interpretation of which retained constituents most plausibly drive the antibacterial, antioxidant, and repellent activities that were experimentally preserved. Molecular docking was used to provide a mechanistic layer that complements the experimental biological observations by identifying whether the major volatile constituents can establish plausible stabilizing interactions within structurally characterized protein pockets related to repellency, antioxidant behavior and antibacterial activity. The selection of the four proteins was guided by this biological rationale: 3N7H represents an insect odorant-binding protein model relevant to volatile recognition and repellent behavior; 3NVY represents xanthine oxidase, an oxidoreductase associated with reactive oxygen species generation and therefore useful for interpreting antioxidant-related activity; and 4URM and 8BN6 represent DNA gyrase B ATPase domains from *Staphylococcus aureus* and *Pseudomonas aeruginosa*, respectively, providing Gram-positive and Gram-negative antibacterial targets consistent with the microbiological assays [68,69]. Because geranial, neral, β-myrcene and geraniol are small monoterpenes, their docking scores are not expected to match those of larger optimized co-crystallized inhibitors; instead, the analysis focuses on relative affinity trends, pose stability and the chemical logic of the observed interactions.

The docking scores in Table 8 show that the co-crystallized ligands bind more strongly than the essential oil constituents, which is expected because the reference ligands are larger, structurally optimized molecules occupying more extensive regions of the binding pockets. Among the selected constituents, however, clear structure-dependent trends are observed. Geraniol gives the best score against 3N7H (−6.4 kcal/mol), followed closely by geranial and neral (−6.3 kcal/mol), supporting the importance of both hydrophobic terpene shape complementarity and a polar oxygen atom for the insect odorant-binding pocket. For 3NVY, geranial and geraniol display the most favorable affinities (−6.1 kcal/mol), indicating that either a carbonyl acceptor or a hydroxyl donor/acceptor can stabilize the xanthine oxidase pocket when coupled to a hydrophobic monoterpene chain. Against the bacterial gyrase targets, the scores are more modest but remain consistent with plausible weak-to-moderate binding: neral is slightly preferred in 4URM (−5.8 kcal/mol), whereas geraniol is preferred in 8BN6 (−5.9 kcal/mol). β-myrcene is consistently the weakest ligand across all targets, reflecting the absence of hydrogen-bonding functionality and indicating that hydrophobic contacts alone are insufficient to maximize binding within these pockets. Overall, the docking results reinforce the biological relevance of oxygenated monoterpenes as the most interactive constituents of the essential oil.

**Figure 5:**
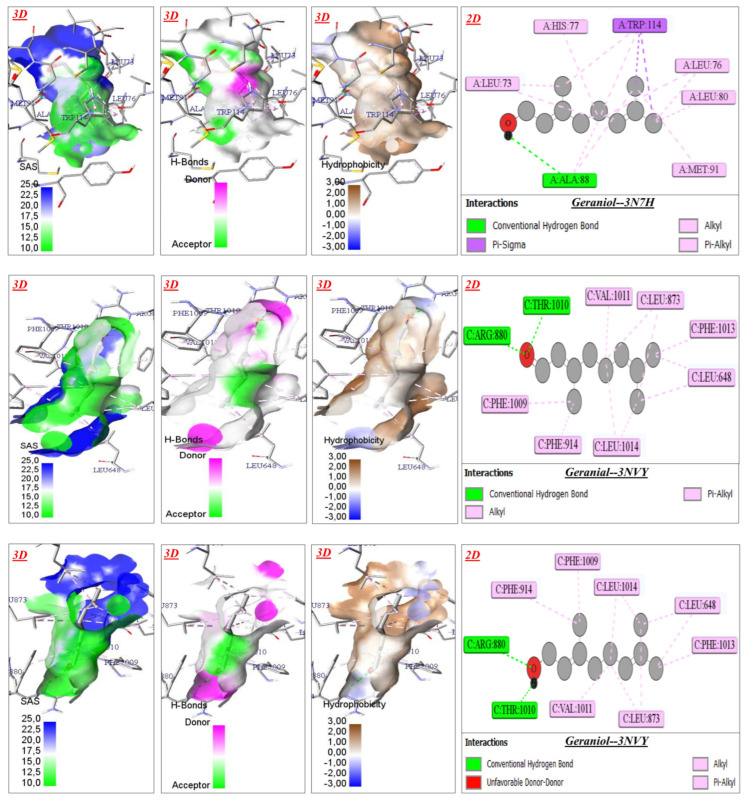
Three-dimensional surface analysis and two-dimensional interaction maps of the most favorable docking complexes selected from Table 8: geraniol-3N7H, geranial-3NVY, and geraniol-3NVY. The 3D panels show solvent-accessible surface (SAS), hydrogen-bond donor/acceptor mapping, and hydrophobicity, while the 2D diagrams identify the main polar and hydrophobic contacts.

Figure 5 provides structural support for the affinity trends observed in Table 8. In the geraniol-3N7H complex, the solvent-accessible surface map shows that the ligand is deeply accommodated within an elongated cavity, with the hydrophobic terpene skeleton buried against an apolar wall and the hydroxyl group oriented toward a polar anchoring zone. The hydrogen-bond surface highlights this localized complementarity, and the 2D interaction map identifies a conventional hydrogen bond with A:ALA88, together with pi-sigma, alkyl and pi-alkyl contacts involving A:TRP114, A:HIS77, A:LEU73, A:LEU76, A:LEU80 and A:MET91. This dual stabilization, combining one directional hydrogen-bond anchor with extensive hydrophobic packing, rationalizes the strongest 3N7H score obtained for geraniol [70,71,72].

For geranial-3NVY, the 3D SAS representation indicates a well-embedded pose within the xanthine oxidase binding channel. The aldehyde oxygen acts as a polar acceptor and is positioned toward the donor-rich region represented by C:ARG880 and C:THR1010, which appear as conventional hydrogen-bond partners in the 2D diagram. Simultaneously, the conjugated hydrocarbon chain aligns along a hydrophobic/aromatic surface formed by residues including C:PHE1009, C:PHE914, C:VAL1011, C:LEU1014, C:LEU873, C:LEU648 and C:PHE1013 [73,74,75,76]. The hydrophobicity panel is therefore highly consistent with the 2D contact network: a polar carbonyl anchor fixes the ligand orientation, while the terpene backbone gains stabilization through van der Waals and pi-alkyl interactions.

The geraniol-3NVY complex exhibits a closely related binding logic but with a hydroxyl-mediated interaction pattern. The hydrogen-bond map shows that the alcohol group remains close to the C:ARG880/C:THR1010 polar region, and the 2D diagram confirms conventional hydrogen bonding with these residues. Although one unfavorable donor–donor contact is indicated, the penalty appears to be compensated by the same hydrophobic enclosure observed for geranial, involving aromatic and aliphatic residues such as C:PHE914, C:PHE1009, C:LEU1014, C:LEU648, C:PHE1013, C:VAL1011 and C:LEU873. Thus, the 3D SAS, hydrogen-bond and hydrophobicity surfaces collectively demonstrate that the highest-ranked complexes are stabilized by a common pharmacophoric pattern: a small oxygenated polar head provides anchoring specificity, whereas the flexible monoterpene chain supplies broad hydrophobic complementarity within the binding pocket. It should be emphasized that these docking and ADMET results are computational predictions intended to generate mechanistic hypotheses consistent with the experimental data; they do not constitute experimental confirmation of target engagement. Enzyme-inhibition assays (e.g., DNA gyrase ATPase and xanthine oxidase inhibition), odorant-binding-protein fluorescence assays, and pharmacokinetic measurements would be required to validate the predicted interactions, and these are identified as priorities for future work.

## 5. Conclusions

This study indicates that, under the conditions tested, gamma irradiation at 5–25 kGy did not cause statistically detectable changes in the measured bioactivities of Cymbopogon citratus essential oil. The citral-dominant chemotype was retained, with geranial and neral remaining the principal constituents and only minor shifts among low-abundance volatiles. Antibacterial activity, antioxidant capacity, and repellency against Tribolium confusum were maintained after irradiation, supporting the potential compatibility of decontamination-level gamma treatment with lemongrass-oil functionality. The docking and ADMET results provide only predictive mechanistic hypotheses, not proof of target engagement or safety. Future work should verify these findings using replicate GC-MS injections, per-dose yield statistics, enzyme- or receptor-level validation, expanded toxicity testing, and larger-scale storage or food-matrix trials.

## Figures and Tables

**Table 1:** Chemical composition of Cymbopogon citratus essential oil at different gamma irradiation doses. RT, retention time; LRI, linear retention index; ΔLRI, difference between the experimental and the selected literature LRI values. Experimental LRIs were calculated from the C8–C25 n-alkane series, and the literature LRIs are selected reference values for DB-5-type non-polar columns; all values are shown as rounded integer indices, and the experimental/literature deviations remained within the usual ±10-unit acceptance range. Compound assignments were retained only when the EI mass spectra, chromatographic elution order, and LRI behavior were mutually consistent. Trace constituents below 0.2% should be interpreted cautiously because replicate GC-MS injections were not available to verify cross-replicate detection consistency. The farnesol-isomer assignment is reported as putative because the available GC-MS/LRI evidence did not resolve the exact isomer.

No	Compound	RT (min)	LRI (exp.)	LRI (lit.)	ΔLRI	0 kGy	5 kGy	15 kGy	25 kGy
1	α-Pinene	8.70	934	932	+2	0.93	0.91	0.99	0.99
2	Camphene	9.12	948	951	−3	0.57	0.55	0.53	0.51
3	6-Methyl-5-hepten-2-one	10.64	988	984	+4	1.65	1.73	1.84	1.97
4	β-Myrcene	10.79	992	988	+4	18.80	18.62	18.34	18.13
5	Limonene	11.72	1029	1031	−2	1.24	1.20	1.16	1.12
6	1,8-Cineole (eucalyptol)	12.14	1034	1030	+4	0.14	0.16	0.19	0.20
7	Acetophenone	13.25	1067	1064	+3	0.27	0.30	0.33	0.37
8	Linalool	14.78	1100	1096	+4	0.63	0.60	0.59	0.57
9	Citronellal	16.64	1154	1150	+4	0.51	0.49	0.48	0.46
10	Carveol	18.96	1226	1221	+5	0.29	0.30	0.33	0.35
11	Neral	19.90	1237	1235	+2	28.96	28.89	28.75	28.45
12	Geraniol	20.42	1257	1253	+4	5.20	5.33	5.71	6.12
13	Geranial	20.96	1268	1272	−4	34.47	34.30	33.99	33.75
14	Citronellyl formate	21.20	1279	1274	+5	0.44	0.42	0.38	0.37
15	Neryl formate	21.35	1290	1294	−4	0.12	0.11	0.11	0.11
16	2-Undecanone	21.56	1295	1291	+4	0.46	0.56	0.57	0.60
17	Geranyl formate	21.83	1301	1306	−5	1.83	1.77	1.73	1.72
18	2-Tridecanone	27.96	1497	1490	+7	0.41	0.46	0.51	0.56
19	Nerolidol	29.05	1561	1565	−4	0.38	0.31	0.33	0.36
20	Caryophyllene oxide	29.40	1580	1586	−6	1.20	1.28	1.37	1.47
21	Putative farnesol isomer	30.57	1719	1725	−6	0.15	0.16	0.18	0.18
Total identified (%)						98.65	98.45	98.41	98.36

**Table 2:** Effect of different irradiation doses on the inhibition-zone diameters (mm) of *Cymbopogon citratus* essential oil against tested microbial strains.

Microbial Strain	0 kGy	5 kGy	15 kGy	25 kGy	Positive Control
*Pseudomonas aeruginosa* (−)	17.4 ± 2.8	17.9 ± 1.9	16.9 ± 2.4	17.6 ± 2.7	19.6 ± 0.8 (GEN)
*Klebsiella pneumoniae* (−)	16.7 ± 1.8	16.8 ± 1.7	17.2 ± 2.4	16.5 ± 1.7	18.9 ± 0.9 (GEN)
*Staphylococcus aureus* (+)	19.2 ± 2.1	18.6 ± 2.5	19.6 ± 3.1	17.8 ± 2.6	21.8 ± 1.0 (VAN)
*Bacillus subtilis* (+)	20.1 ± 2.8	19.5 ± 2.4	19.8 ± 1.6	20.3 ± 1.5	22.4 ± 0.7 (VAN)
*Streptococcus* spp. (+)	21.5 ± 1.8	22.1 ± 2.5	21.8 ± 1.7	22.3 ± 2.6	23.1 ± 1.1 (VAN)

Values are expressed as mean ± standard deviation (*n* = 4). No statistically significant differences were found between values within each row, according to Tukey’s multiple range test (*p* ≥ 0.05). (+) Gram-positive; (−) Gram-negative. Positive controls: GEN, gentamicin (10 µg); VAN, vancomycin (30 µg). The ATCC reference strains showed the expected susceptibility to their control antibiotics; the Streptococcus spp. clinical isolate showed inhibition by vancomycin under these assay conditions, but was not assigned a categorical breakpoint interpretation.

**Table 3:** Minimum inhibitory concentration (µg/mL) of *Cymbopogon citratus* essential oil against different microbial strains at different irradiation doses.

Microbial Strains	Irradiation Dose (kGy)			
	0 kGy	5 kGy	15 kGy	25 kGy
*Pseudomonas aeruginosa*	100	100	100	100
*Klebsiella pneumoniae*	100	100	100	100
*Staphylococcus aureus*	75	75	75	75
*Bacillus subtilis*	75	75	75	75
*Streptococcus* spp.	75	75	75	75

Values are the modal MIC obtained from quadruplicate determinations (*n* = 4); identical values were recorded across all replicates. MIC endpoints were summarized as modal values and were not subjected to inferential statistical testing.

**Table 4:** Minimum bactericidal concentration (µg/mL) of *Cymbopogon citratus* essential oil against different microbial strains at different irradiation doses.

Microbial Strains	Irradiation Dose (kGy)			
	0 kGy	5 kGy	15 kGy	25 kGy
*Pseudomonas aeruginosa*	150	150	150	150
*Klebsiella pneumoniae*	150	150	150	150
*Staphylococcus aureus*	125	125	125	125
*Bacillus subtilis*	125	125	125	125
*Streptococcus* spp.	125	125	125	125

Values are the modal MBC obtained from quadruplicate determinations (*n* = 4); identical values were recorded across all replicates. MBC endpoints were summarized as modal values and were not subjected to inferential statistical testing.

**Table 5:** Antioxidant activity (IC_50_, µg/mL) of *Cymbopogon citratus* essential oil at different irradiation doses measured by DPPH, ABTS, and FRAP assays.

Antioxidant Assay	Irradiation Dose (kGy)			
	0 kGy	5 kGy	15 kGy	25 kGy
DPPH	695.8 ± 10.2	702.5 ± 9.4	697.4 ± 6.7	688.1 ± 9.8
ABTS	21.5 ± 2.4	20.6 ± 3.4	19.8 ± 3.1	18.4 ± 2.7
FRAP	512.6 ± 8.4	516.7 ± 10.2	504.8 ± 8.6	514.2 ± 7.1

Values are expressed as mean ± standard deviation (*n* = 4). No statistically significant differences were found between values within each row, according to Tukey’s multiple range test (*p* ≥ 0.05). DPPH, 2,2-diphenyl-1-picrylhydrazyl; ABTS, 2,2′-azino-bis(3-ethylbenzothiazoline-6-sulfonic acid); FRAP, ferric reducing antioxidant power.

**Table 6:** Repellent effect of non-irradiated essential oil of *Cymbopogon citratus* against adults of *Tribolium confusum*.

Concentration (µL/cm^2^)	Exposure Duration (h)					F	*p*-Value	AR (%)
	0.5	1	2	4	24			
0.031	65 ± 2.5 ^aA^	59 ± 3.9 ^aA^	52 ± 7.1 ^aA^	41 ± 12.4 ^aA^	28 ± 16.5 ^aA^	2.18	0.108	49
0.062	80 ± 5.6 ^abA^	85 ± 5.3 ^bA^	72 ± 6.1 ^aA^	73 ± 7.9 ^aA^	64 ± 8.6 ^abA^	2.41	0.084	74
0.125	89 ± 4.0 ^bA^	89 ± 1.6 ^bA^	95 ± 1.3 ^bA^	93 ± 2.1 ^bA^	92 ± 2.5 ^bA^	0.91	0.475	92
F (2,12)	8.23	18.04	15.23	10.26	8.75			
*p*-value	0.006	<0.001	0.001	0.003	0.005			

Values expressed as mean of percentage of repellency (PR) ± standard error for five replicates using 30 adults each. Results are considered significantly different when the lowercase and uppercase letters differ at *p* < 0.05 across concentrations and exposure durations, respectively. AR, average repellency over the experiment.

**Table 7:** SwissADME- and pkCSM-derived ADME descriptors of the selected major constituents of *Cymbopogon citratus* essential oil.

Parameter	Geranial	Neral	β-Myrcene	Geraniol
Section 1: Physicochemical properties				
Molecular weight (g/mol)	152.23	152.23	136.23	154.25
H-bond acceptors	1	1	0	1
H-bond donors	0	0	0	1
Rotatable bonds	4	4	5	4
Molar refractivity	49.44	49.44	48.76	50.40
TPSA (Å^2^)	17.07	17.07	0	20.23
Consensus Log P (o/w)	2.71	2.71	3.55	2.74
Log S (w)	−1.96	−1.96	−2.79	−1.84
Section 2: Drug-likeness and oral bioavailability				
GI absorption	High	High	Low	High
Lipinski violations	0	0	0	0
Veber violations	0	0	0	0
Bioavailability score	0.55	0.55	0.55	0.55
Intestinal absorption (human, %)	95.317	95.317	95.124	92.788
Section 3: BBB and CNS penetration				
BBB permeability	0.626	0.626	0.812	0.606
CNS permeability	−1.986	−1.986	−1.965	−2.159
Section 4: Metabolic and transporter interactions				
CYP2D6 inhibitor	No	No	No	No
CYP3A4 inhibitor	No	No	No	No
CYP2D6 substrate	No	No	No	No
CYP3A4 substrate	No	No	No	No
Section 5: Excretion and clearance				
Renal OCT2 substrate	No	No	No	No
Total clearance (log mL/min/kg)	0.376	0.376	0.460	0.437

**Table 8:** Molecular docking binding affinities (kcal/mol) of the selected major constituents and native/co-crystallized ligands against 3N7H, 3NVY, 4URM, and 8BN6.

Compound	3N7H	3NVY	4URM	8BN6
Geranial	−6.3	−6.1	−5.6	−5.7
Neral	−6.3	−5.7	−5.8	−5.8
β-Myrcene	−5.9	−5.3	−5.0	−5.2
Geraniol	−6.4	−6.1	−5.7	−5.9
Co-3N7H	−7.6	N/A	N/A	N/A
Co-3NVY	N/A	−8.7	N/A	N/A
Co-4URM	N/A	N/A	−10.3	N/A
Co-8BN6	N/A	N/A	N/A	−9.3

N/A: not applicable.

## Data Availability

All summarized data supporting the reported results are presented within the article. Raw chromatograms, n-alkane calibration/LRI calculation files, EO-yield worksheets, antimicrobial positive-control records, and additional data generated during the study are available from the corresponding author on reasonable request.

## Supplementary Materials

The following supporting information can be downloaded at: https://www.mdpi.com/article/10.3390/microorganisms14071417/s1, Figure S1: Exploratory hierarchical cluster analysis (HCA; average linkage, Euclidean distance; row-standardized area percentages) of the GC-MS volatile profile of Cymbopogon citratus essential oil across the four gamma irradiation doses (0, 5, 15, and 25 kGy). The display order of dose columns follows dendrogram clustering rather than numerical dose order. The apparent low-/high-dose grouping is interpreted as trace-level minor-compound noise within single-injection profiles and was not statistically tested. Because GC-MS replicate injections were not available, the HCA is intended only as a descriptive visualization and not as evidence of treatment separation.

## Abbreviations

The following abbreviations are used in this manuscript: EO, essential oil; GC-MS, gas chromatography–mass spectrometry; EI, electron impact; LRI/RI, linear retention index; NIST, National Institute of Standards and Technology; MHA, Mueller–Hinton agar; CAMHB, cation-adjusted Mueller–Hinton broth; CFU, colony-forming units; MIC, minimum inhibitory concentration; MBC, minimum bactericidal concentration; CLSI, Clinical and Laboratory Standards Institute; EUCAST, European Committee on Antimicrobial Susceptibility Testing; DPPH, 2,2-diphenyl-1-picrylhydrazyl; ABTS, 2,2′-azino-bis(3-ethylbenzothiazoline-6-sulfonic acid); FRAP, ferric reducing antioxidant power; TPTZ, 2,4,6-tris(2-pyridyl)-s-triazine; PR, percentage of repellency; AR, average repellency; ANOVA, analysis of variance; SD, standard deviation; SE, standard error; ADME(T), absorption, distribution, metabolism, excretion (and toxicity); TPSA, topological polar surface area; Log P, partition coefficient; GI, gastrointestinal; BBB, blood–brain barrier; CNS, central nervous system; CYP, cytochrome P450; OCT2, organic cation transporter 2; P-gp, P-glycoprotein; SAS, solvent-accessible surface; RMSD, root-mean-square deviation; PDB, Protein Data Bank; OBP, odorant-binding protein.
