# Supplementary material for: Phytochemical, Antimicrobial, Insect-Repellent, and Molecular Docking Profiles of Gamma-Irradiated Cymbopogon citratus Essential Oil
Source: Microorganisms. 2026 Jun 28;14(7):1417. doi: 10.3390/microorganisms14071417 (PMC13414017; doi:10.3390/microorganisms14071417)
Supplement: Supplementary file 1 [file microorganisms-14-01417-s001.zip › microorganisms-4337112-supplementary.pdf]

Supplementary Materials

Figure S1. Exploratory hierarchical cluster analysis of GC-MS volatile profiles.

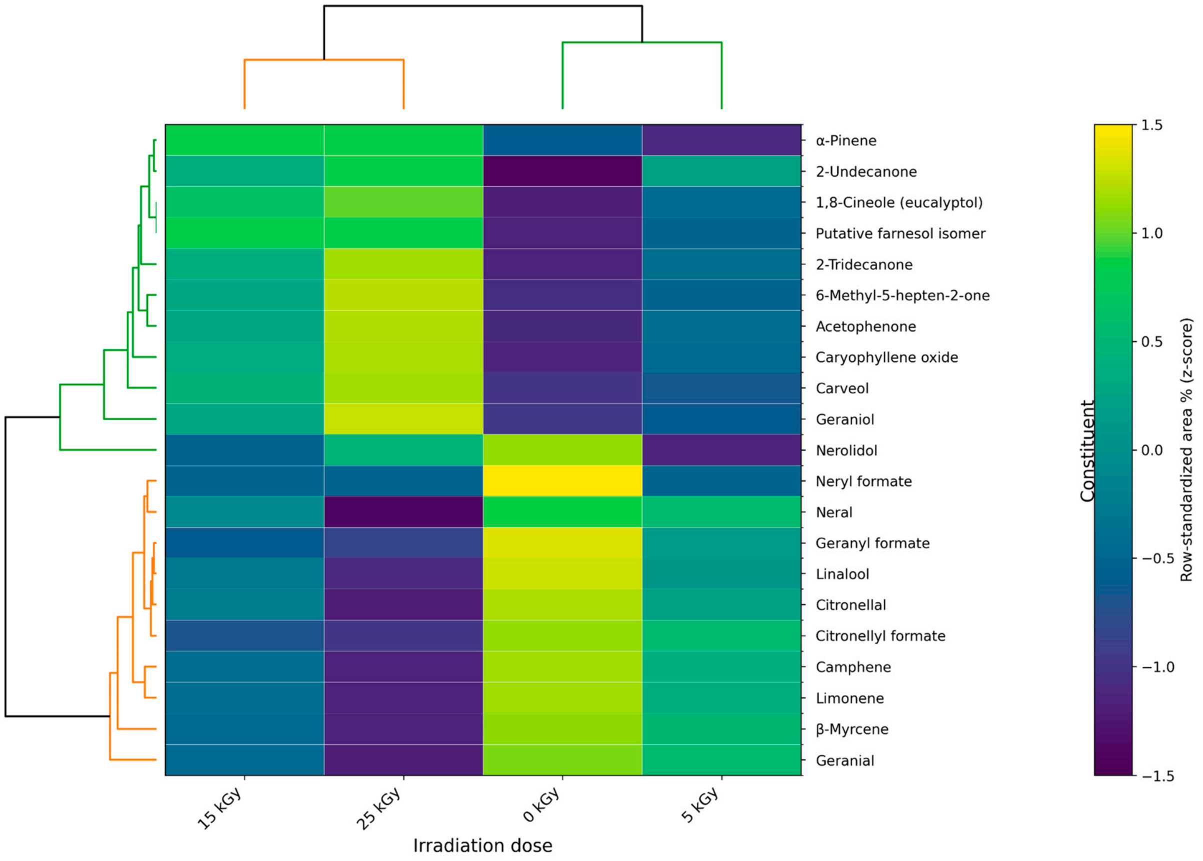

Figure S1. Exploratory hierarchical cluster analysis (HCA; average linkage, Euclidean distance; row-standardized area percentages) of the GC-MS volatile profile of Cymbopogon citratus essential oil across the four gamma irradiation doses (0, 5, 15, and 25 kGy). The display order of dose columns follows dendrogram clustering rather than numerical dose order. The apparent low-/high-dose grouping is interpreted as trace-level minor-compound noise within single-injection profiles and was not statistically tested. Because GC-MS replicate injections were not available, the HCA is intended only as a descriptive visualization and not as evidence of treatment separation.
